# Benzyl *N*′-(1-methyl-1*H*-indol-3-yl­methyl­idene)hydrazinecarbodithio­ate

**DOI:** 10.1107/S1600536808039330

**Published:** 2008-11-29

**Authors:** Hamid Khaledi, Hapipah Mohd Ali, Seik Weng Ng

**Affiliations:** a Department of Chemistry, University of Malaya, 50603 Kuala Lumpur, Malaysia

## Abstract

The *N*′-(1-methyl-1*H*-indol-3-ylmethyl­idene)hydrazine­carbo­dithio­ate portion of the title mol­ecule, C_18_H_17_N_3_S_2_, is nearly planar; this unit and the phenyl ring subtend an angle of 112.9 (2)° at the methyl­ene C atom.

## Related literature

For the structure of *S*-benzyl *N*-1-(1*H*-indol-3-ylmethyl­idenehydrazinecarbodithio­ate, see: Khaledi *et al.* (2008 ▶).
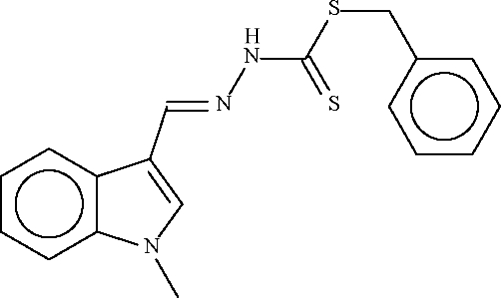


## Experimental

### 

#### Crystal data

C_18_H_17_N_3_S_2_
*M* *_r_* = 339.47
Monoclinic, 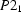

*a* = 10.6111 (4) Å
*b* = 6.1134 (2) Å
*c* = 13.4961 (4) Å
β = 111.934 (2)°
*V* = 812.12 (5) Å^3^
*Z* = 2
Mo *K*α radiation
μ = 0.33 mm^−1^
*T* = 100 (2) K
0.30 × 0.10 × 0.02 mm

#### Data collection

Bruker SMART APEX diffractometer
Absorption correction: multi-scan (*SADABS*; Sheldrick, 1996 ▶) *T*_min_ = 0.908, *T*_max_ = 0.993
5513 measured reflections
3277 independent reflections
2802 reflections with *I* > 2σ(*I*)
*R*_int_ = 0.027

#### Refinement

*R*[*F* ^2^ > 2σ(*F* ^2^)] = 0.037
*wR*(*F* ^2^) = 0.087
*S* = 1.03
3277 reflections
213 parameters
2 restraints
H atoms treated by a mixture of independent and constrained refinement
Δρ_max_ = 0.28 e Å^−3^
Δρ_min_ = −0.28 e Å^−3^
Absolute structure: Flack (1983 ▶), 1261 Friedel pairs
Flack parameter: 0.01 (8)

### 

Data collection: *APEX2* (Bruker, 2007 ▶); cell refinement: *SAINT* (Bruker, 2007 ▶); data reduction: *SAINT*; program(s) used to solve structure: *SHELXS97* (Sheldrick, 2008 ▶); program(s) used to refine structure: *SHELXL97* (Sheldrick, 2008 ▶); molecular graphics: *X-SEED* (Barbour, 2001 ▶); software used to prepare material for publication: *publCIF* (Westrip, 2008 ▶).

## Supplementary Material

Crystal structure: contains datablocks global, I. DOI: 10.1107/S1600536808039330/tk2334sup1.cif

Structure factors: contains datablocks I. DOI: 10.1107/S1600536808039330/tk2334Isup2.hkl

Additional supplementary materials:  crystallographic information; 3D view; checkCIF report

## Figures and Tables

**Fig. 1.**
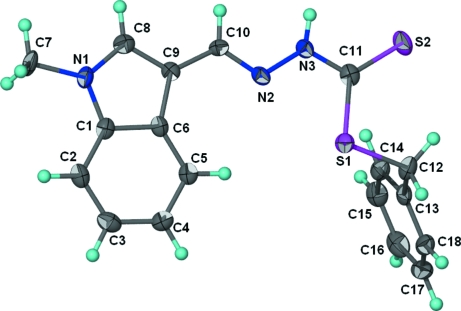
Thermal ellipsoid plot (Barbour, 2001) of C18H17N2S3 at the 70% probability level. Hydrogen atoms are drawn as spheres of arbitrary radius.

## supplementary crystallographic information

### Comment

(type here to add)

### Experimental

*N*-Methylindole-3-carbaldehyde (1.59 g, 10 mmol) and *S*-benzyl dithiocarbazate (1.98 g, 10 mmol) were heated in ethanol (60 ml) for 1 h. Several drops of acetic acid were added. The solution yielded a solid on cooling. This was recrystallized from DMSO.

### Refinement

Hydrogen atoms were placed at calculated positions (C–H 0.95–0.99 Å) and were treated as riding on their parent carbon atoms, with *U*(H) set to 1.2 times *U*_eq_(C), 1.5 for methyl-C. The amino H-atom was located in a difference Fourier map, and was refined with a distance restraint of N–H 0.88±0.01 Å; it does not form a hydrogen bond.

### Crystal data

**Table:**

C_18_H_17_N_3_S_2_	*F*_000_ = 356
*M**_r_* = 339.47	*D*_x_ = 1.388 Mg m^−^^3^
Monoclinic, *P*2_1_	Mo *K*α radiation λ = 0.71073 Å
Hall symbol: P 2yb	Cell parameters from 1721 reflections
*a* = 10.6111 (4) Å	θ = 3.1–27.1º
*b* = 6.1134 (2) Å	µ = 0.33 mm^−^^1^
*c* = 13.4961 (4) Å	*T* = 100 (2) K
β = 111.934 (2)º	Plate, yellow
*V* = 812.12 (5) Å^3^	0.30 × 0.10 × 0.02 mm
*Z* = 2	

### Data collection

**Table:**

Bruker SMART APEX diffractometer	3277 independent reflections
Radiation source: fine-focus sealed tube	2802 reflections with *I* > 2σ(*I*)
Monochromator: graphite	*R*_int_ = 0.027
*T* = 100(2) K	θ_max_ = 27.5º
ω scans	θ_min_ = 1.6º
Absorption correction: Multi-scan(SADABS; Sheldrick, 1996)	*h* = −13→13
*T*_min_ = 0.908, *T*_max_ = 0.993	*k* = −6→7
5513 measured reflections	*l* = −17→17

### Refinement

**Table:**

Refinement on *F*^2^	Hydrogen site location: constr
Least-squares matrix: full	H atoms treated by a mixture of independent and constrained refinement
*R*[*F*^2^ > 2σ(*F*^2^)] = 0.037	*w* = 1/[σ^2^(*F*_o_^2^) + (0.0366*P*)^2^ + 0.212*P*] where *P* = (*F*_o_^2^ + 2*F*_c_^2^)/3
*wR*(*F*^2^) = 0.087	(Δ/σ)_max_ = 0.001
*S* = 1.03	Δρ_max_ = 0.28 e Å^−^^3^
3277 reflections	Δρ_min_ = −0.28 e Å^−^^3^
213 parameters	Extinction correction: none
2 restraints	Absolute structure: Flack (1983), 1261 Friedel pairs
Primary atom site location: structure-invariant direct methods	Flack parameter: 0.01 (8)
Secondary atom site location: difference Fourier map	

### Fractional atomic coordinates and isotropic or equivalent isotropic displacement parameters (Å^2^)

**Table:**

	*x*	*y*	*z*	*U*_iso_*/*U*_eq_	
S1	0.33191 (7)	0.50000 (12)	0.67816 (5)	0.01960 (16)	
S2	0.48123 (7)	0.86601 (13)	0.61270 (5)	0.02642 (18)	
N1	0.2902 (2)	0.7662 (4)	1.16590 (16)	0.0202 (5)	
N2	0.3791 (2)	0.7502 (4)	0.85695 (16)	0.0181 (5)	
N3	0.4323 (2)	0.8477 (4)	0.78860 (16)	0.0183 (5)	
H3	0.480 (3)	0.967 (3)	0.796 (2)	0.035 (9)*	
C1	0.2337 (3)	0.5789 (5)	1.1079 (2)	0.0178 (6)	
C2	0.1584 (3)	0.4147 (5)	1.1303 (2)	0.0215 (6)	
H2	0.1422	0.4143	1.1950	0.026*	
C3	0.1075 (3)	0.2515 (5)	1.0548 (2)	0.0236 (6)	
H3A	0.0531	0.1389	1.0668	0.028*	
C4	0.1347 (3)	0.2496 (5)	0.9610 (2)	0.0215 (6)	
H4	0.0983	0.1354	0.9106	0.026*	
C5	0.2133 (3)	0.4101 (4)	0.9399 (2)	0.0193 (6)	
H5	0.2326	0.4051	0.8767	0.023*	
C6	0.2637 (3)	0.5795 (4)	1.01374 (19)	0.0166 (6)	
C7	0.2743 (3)	0.8324 (5)	1.2645 (2)	0.0261 (7)	
H7A	0.3162	0.9761	1.2867	0.039*	
H7B	0.3186	0.7249	1.3206	0.039*	
H7C	0.1774	0.8405	1.2524	0.039*	
C8	0.3524 (3)	0.8815 (5)	1.11224 (19)	0.0201 (6)	
H8	0.3981	1.0166	1.1357	0.024*	
C9	0.3409 (3)	0.7767 (4)	1.01840 (19)	0.0168 (6)	
C10	0.3932 (2)	0.8586 (5)	0.94249 (18)	0.0166 (5)	
H10	0.4392	0.9952	0.9551	0.020*	
C11	0.4177 (3)	0.7519 (5)	0.6953 (2)	0.0197 (6)	
C12	0.3161 (3)	0.4207 (5)	0.54473 (19)	0.0229 (6)	
H12A	0.3840	0.5019	0.5251	0.028*	
H12B	0.3366	0.2627	0.5445	0.028*	
C13	0.1765 (3)	0.4642 (5)	0.46213 (19)	0.0192 (6)	
C14	0.1145 (3)	0.6657 (5)	0.4536 (2)	0.0220 (6)	
H14	0.1606	0.7800	0.5010	0.026*	
C15	−0.0137 (3)	0.7043 (5)	0.3773 (2)	0.0258 (7)	
H15	−0.0550	0.8438	0.3726	0.031*	
C16	−0.0816 (3)	0.5379 (6)	0.3078 (2)	0.0295 (8)	
H16	−0.1693	0.5638	0.2552	0.035*	
C17	−0.0220 (3)	0.3353 (5)	0.3150 (2)	0.0280 (7)	
H17	−0.0686	0.2212	0.2677	0.034*	
C18	0.1073 (3)	0.2984 (5)	0.3919 (2)	0.0240 (7)	
H18	0.1486	0.1589	0.3966	0.029*	

### Atomic displacement parameters (Å^2^)

**Table:**

	*U*^11^	*U*^22^	*U*^33^	*U*^12^	*U*^13^	*U*^23^
S1	0.0229 (3)	0.0209 (4)	0.0149 (3)	−0.0023 (3)	0.0070 (2)	0.0006 (3)
S2	0.0317 (4)	0.0286 (4)	0.0252 (3)	−0.0013 (4)	0.0179 (3)	0.0058 (3)
N1	0.0225 (12)	0.0243 (13)	0.0158 (10)	0.0007 (10)	0.0094 (9)	−0.0026 (9)
N2	0.0191 (11)	0.0187 (13)	0.0176 (10)	−0.0022 (10)	0.0082 (9)	0.0036 (9)
N3	0.0232 (12)	0.0166 (13)	0.0178 (10)	−0.0018 (10)	0.0109 (9)	0.0023 (10)
C1	0.0179 (14)	0.0190 (15)	0.0159 (12)	0.0050 (12)	0.0056 (11)	0.0017 (11)
C2	0.0210 (14)	0.0248 (17)	0.0208 (13)	0.0049 (12)	0.0103 (11)	0.0051 (11)
C3	0.0217 (14)	0.0195 (16)	0.0298 (14)	−0.0007 (13)	0.0097 (12)	0.0052 (12)
C4	0.0225 (14)	0.0195 (15)	0.0208 (13)	−0.0007 (12)	0.0062 (11)	−0.0012 (12)
C5	0.0192 (13)	0.0213 (16)	0.0178 (12)	0.0031 (12)	0.0072 (11)	0.0025 (11)
C6	0.0145 (13)	0.0187 (15)	0.0169 (12)	0.0041 (11)	0.0063 (10)	0.0031 (11)
C7	0.0369 (17)	0.0297 (18)	0.0160 (12)	0.0016 (14)	0.0148 (12)	−0.0056 (12)
C8	0.0173 (13)	0.0194 (15)	0.0216 (12)	0.0007 (13)	0.0049 (10)	−0.0003 (12)
C9	0.0156 (13)	0.0182 (15)	0.0157 (12)	0.0003 (11)	0.0048 (10)	0.0016 (11)
C10	0.0180 (13)	0.0136 (13)	0.0168 (11)	−0.0014 (12)	0.0049 (10)	−0.0002 (11)
C11	0.0176 (13)	0.0225 (16)	0.0185 (12)	0.0038 (12)	0.0061 (11)	0.0040 (11)
C12	0.0298 (15)	0.0225 (16)	0.0182 (12)	0.0035 (12)	0.0109 (12)	−0.0016 (11)
C13	0.0230 (13)	0.0242 (17)	0.0130 (11)	−0.0008 (12)	0.0099 (10)	0.0005 (11)
C14	0.0230 (15)	0.0266 (17)	0.0157 (12)	−0.0016 (12)	0.0064 (11)	−0.0021 (11)
C15	0.0281 (16)	0.0256 (17)	0.0254 (14)	0.0045 (13)	0.0122 (12)	0.0060 (13)
C16	0.0244 (15)	0.044 (2)	0.0176 (12)	−0.0029 (14)	0.0055 (11)	0.0064 (13)
C17	0.0353 (17)	0.0324 (19)	0.0169 (12)	−0.0157 (15)	0.0105 (12)	−0.0049 (13)
C18	0.0366 (17)	0.0196 (16)	0.0202 (13)	−0.0043 (13)	0.0159 (13)	−0.0011 (11)

### Geometric parameters (Å, °)

**Table:**

S1—C11	1.759 (3)	C7—H7A	0.9800
S1—C12	1.811 (3)	C7—H7B	0.9800
S2—C11	1.657 (3)	C7—H7C	0.9800
N1—C8	1.347 (3)	C8—C9	1.383 (4)
N1—C1	1.390 (4)	C8—H8	0.9500
N1—C7	1.460 (3)	C9—C10	1.426 (4)
N2—C10	1.290 (3)	C10—H10	0.9500
N2—N3	1.384 (3)	C12—C13	1.508 (4)
N3—C11	1.345 (3)	C12—H12A	0.9900
N3—H3	0.875 (10)	C12—H12B	0.9900
C1—C2	1.386 (4)	C13—C14	1.381 (4)
C1—C6	1.420 (4)	C13—C18	1.394 (4)
C2—C3	1.384 (4)	C14—C15	1.386 (4)
C2—H2	0.9500	C14—H14	0.9500
C3—C4	1.400 (4)	C15—C16	1.389 (4)
C3—H3A	0.9500	C15—H15	0.9500
C4—C5	1.385 (4)	C16—C17	1.378 (5)
C4—H4	0.9500	C16—H16	0.9500
C5—C6	1.398 (4)	C17—C18	1.395 (4)
C5—H5	0.9500	C17—H17	0.9500
C6—C9	1.445 (4)	C18—H18	0.9500
			
C11—S1—C12	102.48 (13)	C9—C8—H8	124.6
C8—N1—C1	108.8 (2)	C8—C9—C10	124.8 (3)
C8—N1—C7	126.3 (3)	C8—C9—C6	106.3 (2)
C1—N1—C7	124.7 (2)	C10—C9—C6	128.9 (2)
C10—N2—N3	115.8 (2)	N2—C10—C9	121.1 (3)
C11—N3—N2	120.3 (2)	N2—C10—H10	119.5
C11—N3—H3	109 (2)	C9—C10—H10	119.5
N2—N3—H3	131 (2)	N3—C11—S2	120.9 (2)
C2—C1—N1	129.5 (2)	N3—C11—S1	112.2 (2)
C2—C1—C6	122.6 (3)	S2—C11—S1	126.94 (17)
N1—C1—C6	108.0 (2)	C13—C12—S1	112.87 (19)
C3—C2—C1	117.4 (2)	C13—C12—H12A	109.0
C3—C2—H2	121.3	S1—C12—H12A	109.0
C1—C2—H2	121.3	C13—C12—H12B	109.0
C2—C3—C4	121.1 (3)	S1—C12—H12B	109.0
C2—C3—H3A	119.4	H12A—C12—H12B	107.8
C4—C3—H3A	119.4	C14—C13—C18	118.6 (2)
C5—C4—C3	121.6 (3)	C14—C13—C12	121.6 (2)
C5—C4—H4	119.2	C18—C13—C12	119.8 (3)
C3—C4—H4	119.2	C13—C14—C15	121.2 (3)
C4—C5—C6	118.6 (2)	C13—C14—H14	119.4
C4—C5—H5	120.7	C15—C14—H14	119.4
C6—C5—H5	120.7	C14—C15—C16	119.7 (3)
C5—C6—C1	118.8 (2)	C14—C15—H15	120.2
C5—C6—C9	135.2 (2)	C16—C15—H15	120.2
C1—C6—C9	106.0 (2)	C17—C16—C15	120.2 (3)
N1—C7—H7A	109.5	C17—C16—H16	119.9
N1—C7—H7B	109.5	C15—C16—H16	119.9
H7A—C7—H7B	109.5	C16—C17—C18	119.7 (3)
N1—C7—H7C	109.5	C16—C17—H17	120.2
H7A—C7—H7C	109.5	C18—C17—H17	120.2
H7B—C7—H7C	109.5	C13—C18—C17	120.7 (3)
N1—C8—C9	110.9 (3)	C13—C18—H18	119.7
N1—C8—H8	124.6	C17—C18—H18	119.7
			
C10—N2—N3—C11	−177.2 (2)	C1—C6—C9—C8	−0.2 (3)
C8—N1—C1—C2	−178.6 (3)	C5—C6—C9—C10	−1.0 (5)
C7—N1—C1—C2	−3.0 (4)	C1—C6—C9—C10	−178.3 (3)
C8—N1—C1—C6	0.3 (3)	N3—N2—C10—C9	179.1 (2)
C7—N1—C1—C6	175.9 (2)	C8—C9—C10—N2	179.5 (3)
N1—C1—C2—C3	176.3 (3)	C6—C9—C10—N2	−2.7 (4)
C6—C1—C2—C3	−2.4 (4)	N2—N3—C11—S2	−179.82 (19)
C1—C2—C3—C4	1.8 (4)	N2—N3—C11—S1	−1.8 (3)
C2—C3—C4—C5	0.0 (4)	C12—S1—C11—N3	175.97 (19)
C3—C4—C5—C6	−1.3 (4)	C12—S1—C11—S2	−6.1 (2)
C4—C5—C6—C1	0.8 (4)	C11—S1—C12—C13	−101.3 (2)
C4—C5—C6—C9	−176.2 (3)	S1—C12—C13—C14	50.8 (3)
C2—C1—C6—C5	1.1 (4)	S1—C12—C13—C18	−129.3 (2)
N1—C1—C6—C5	−177.9 (2)	C18—C13—C14—C15	0.0 (4)
C2—C1—C6—C9	178.9 (2)	C12—C13—C14—C15	179.9 (2)
N1—C1—C6—C9	−0.1 (3)	C13—C14—C15—C16	−0.1 (4)
C1—N1—C8—C9	−0.4 (3)	C14—C15—C16—C17	0.3 (4)
C7—N1—C8—C9	−175.9 (3)	C15—C16—C17—C18	−0.4 (4)
N1—C8—C9—C10	178.6 (2)	C14—C13—C18—C17	−0.2 (4)
N1—C8—C9—C6	0.4 (3)	C12—C13—C18—C17	180.0 (2)
C5—C6—C9—C8	177.1 (3)	C16—C17—C18—C13	0.4 (4)
